# Human Immunodeficiency Virus Type 2: The Neglected Threat

**DOI:** 10.3390/pathogens10111377

**Published:** 2021-10-25

**Authors:** Giancarlo Ceccarelli, Marta Giovanetti, Caterina Sagnelli, Alessandra Ciccozzi, Gabriella d’Ettorre, Silvia Angeletti, Alessandra Borsetti, Massimo Ciccozzi

**Affiliations:** 1 Department of Public Health and Infectious Diseases, Policlinico Umberto I, Sapienza University of Rome, Piazzale Aldo Moro 5, 00185 Rome, Italy; giancarlo.ceccarelli@uniroma1.it (G.C.); gabriella.dettorre@uniroma1.it (G.d.); 2 Laboratório de Flavivírus, Instituto Oswaldo Cruz, Fundação Oswaldo Cruz, Rio de Janeiro 21040-360, Brazil; marta.giovanetti@ioc.fiocruz.br; 3 Laboratório de Genética Celular e Molecular, Universidade Federal de Minas Gerais, Belo Horizonte 31270-901, Brazil; 4 Section of Infectious Diseases, Department of Mental Health and Public Medicine, University of Campania Luigi Vanvitelli, Via L. Armanni 5, 80131 Naples, Italy; caterina.sagnelli@unicampania.it; 5 Unit of Medical Statistics and Molecular Epidemiology, University Campus Bio-Medico of Rome, 00100 Rome, Italy; alessandra.ciccozzi@gmail.com; 6 Unit of Clinical Laboratory Science, University Campus Bio-Medico of Rome, 00100 Rome, Italy; s.angeletti@unicampus.it; 7 National HIV/AIDS Research Center, Istituto Superiore di Sanità, 00100 Rome, Italy; alessandra.borsetti@iss.it

**Keywords:** HIV-2, epidemiology, AIDS

## Abstract

West Africa has the highest prevalence of human immunodeficiency virus (HIV)-2 infection in the world, but a high number of cases has been recognized in Europe, India, and the United States. The virus is less transmissible than HIV-1, with sexual contacts being the most frequent route of acquisition. In the absence of specific antiretroviral therapy, most HIV-2 carriers will develop AIDS. Although, it requires more time than HIV-1 infection, CD4+ T cell decline occurs more slowly in HIV-2 than in HIV-1 patients. HIV-2 is resistant to non-nucleoside reverse transcriptase inhibitors (NNRTIs) and some protease inhibitors. Misdiagnosis of HIV-2 in patients mistakenly considered HIV-1-positive or in those with dual infections can cause treatment failures with undetectable HIV-1 RNA. In this era of global integration, clinicians must be aware of when to consider the diagnosis of HIV-2 infection and how to test for this virus. Although there is debate regarding when therapy should be initiated and which regimen should be chosen, recent trials have provided important information on treatment options for HIV-2 infection. In this review, we focus mainly on data available and on the insight they offer about molecular epidemiology, clinical presentation, antiretroviral therapy, and diagnostic tests of HIV-2 infection.

## 1. Introduction

Human immunodeficiency virus (HIV) has emerged as one of the major challenges for the world, becoming one of the leading causes of mortality and burden over the last decade [1]. It was estimated that approximately 37.6 million people across the globe lived with HIV in 2020. Of these, up to 2 million were infected with HIV-2, although this last prevalence reported was likely an underestimation of the epidemiological situation [2,3]. HIV-1 and HIV-2 originated in non-human primates in West–Central Africa through a process known as zoonosis, probably transferred to humans in the early 20th century but were discovered only in the 1980s of the last century [1]. Although both viruses share similar transmission routes, they differ in regard to their epidemiology and clinical management [4]. Despite these differences, currently, the approach to HIV-2 suffers from a lack of studies dedicated to its specificities and has largely been extrapolated from studies in HIV-1 though notable existing differences. In particular, there is a paucity of epidemiologic surveys, clinical studies, and drug trials designed to address the current limitations in understanding of HIV-2 management due to its lower prevalence compared to HIV-1 and its geographical distribution being more frequent in developing countries [2,3].

The thirty-year investment of resources has made it possible to transform HIV-1 infection from a fatal to a chronic disease, with prolonged survival and good quality of life, in developed countries. Unlike HIV-1, scientific research has not invested the same amount of resources for HIV-2. This disease has therefore remained on the sidelines and currently has all the characteristics to be rightly classified among the “forgotten diseases”, even though it is not yet included in the WHO portfolio of tropical neglected diseases [5,6,7].

In particular, UNAIDS, programmed in 2015, is an ambitious project to counter the worldwide HIV epidemic: this global strategy, known as 90-90-90, had the target to reach (a) 90% of HIV infections diagnosed, (b) 90% of infections already diagnosed on treatment, and (c) the achievement of suppressed viral load in 90% of the infections on treatment in 2020. However, the project was largely focused on HIV-1 management and treatment, while the effort to achieve the same goals for HIV-2 was significantly more limited. New impetus and attention are currently being loudly invoked by the scientific community for the study of HIV-2 and management of affected people [3,8].

In this review, we analyzed the recent progress contributions regarding HIV-2 clinical presentation, antiretroviral therapy, diagnostic tests, molecular epidemiology, and transmission patterns, highlighting how challenging problems appear to improve our knowledge on this forgotten viral pathogen.

## 2. HIV-2 Origin and Infection Worldwide

The existence of HIV-2 was first suspected in 1985 with Senegalese patients, suggesting the existence of a type of HIV with a serological profile more closely related to *Simian immunodeficiency virus* (SIV) than HIV-1, and was later isolated in West Africa in the mid-1980s among people who lived with AIDS [9,10,11,12]. Nevertheless, phylogenetic studies and molecular clock analyses suggest it emerged from SIV-infected sooty mangabey monkeys and has likely crossed species into humans between the 1940–50s, probability in Guinea-Bissau [12,13].

Unlike the global spread of HIV-1, HIV-2 has remained limited over time to specific geographical areas, mainly in the African continent.

Currently, the absolute number of people living with HIV-2 worldwide is unknown and reliable data are missing: this lack of up-to-date data is also observable in the most recent HIV-2 management guidelines which collect and continue to report possibly outdated epidemiological information [9,14]. Last available data showed that HIV-2 is endemic in West Africa, where it probably infects 1–2 million people, while clusters or sporadic cases are described among other countries [10,13,14,15]. Historically, the highest prevalence was reported in Guinea-Bissau, including around 8% of the adult population and up to 20% in the subgroup of individuals over the age of 40 [16]. At the turn of the 20th and 21st centuries, other West African countries significantly affected by HIV-2 were Senegal, Niger, Burkina Faso, Ghana, Guinea, Benin, Liberia, Togo, Angola, Mozambique, and São Tomé (all with a prevalence of >1%) [10,15,17,18]. In the same geographical area, between 10 and 20% of HIV infections included HIV-2 with doubly infected or reactive HIV-1/HIV-2 individuals [19]. 

Despite the lack of up-to-date data, currently, the epidemiology previously reported for HIV-2 seems to be shifting towards an overall contraction in endemic countries: in fact, its global prevalence, which had peaked in 1980, is progressively decreasing due to multiple factors not yet well characterized but mainly attributable to lower transmission efficiency, decreasing HIV-2 fitness, and competitive exclusion of HIV-1 [10,15,20,21,22,23]. The most up-to-date available data reports that prevalence in Guinea-Bissau, Senegal, Gambia, Sierra, Leone, and the Ivory Coast ranges from 1 to 5%, while in all other West African countries, including Cape Verde, it is less than 1% [20].

Apart from the endemic area, an intermediate incidence was previously reported in North America for the large presence of West African migrants. It is also already widespread in Brazil, in the Goa region (India), and in a number of European countries. In Europe, Portugal and France have the highest prevalence, but also Belgium and Spain report a significant number of cases. In Italy, this neglected infection has been recently highlighted because of the migratory flow from different countries [10,15,24,25,26,27,28].

The current number of HIV-2 cases in the extra-African regions is probably underestimated because of globalization, widespread immigration to developed countries, and trade ties. These latter factors, along with international travel, have contributed to the global spread of HIV-2 infection [10,15,29,30].

Based on currently available information, in Europe, France and Portugal reported the highest prevalence (around 1000 and 2000 cases, respectively) [20,31,32]. Between 2010 and 2017, the CDC reported 198 cases of HIV-2 in the United States of America [33]. On the Asian continent, India is the country most affected by the problem, with a prevalence ranging from 0.14% to 2.1% mainly in the western and southern regions [34].

## 3. HIV-2 Genetic Diversity and Molecular Epidemiology

HIV-2 virulence properties vary significantly and range from relative attenuation in certain individuals to high-level pathogenicity in others. These differences in clinical manifestations may be determined by the genetic diversity of infecting virus strains. Recombination is an important phenomenon for viral diversification that allows the virus to evade the host immune system and antiretroviral treatment [14]. The process of the genetic recombination of viral genomes of different subtypes is also the basis for the emergence of the so-called “mosaic” strains designated as circulating recombinant forms (CRF). Although the viral subtype is probably not a major determinant of disease progression and it has no impact on HIV-2 molecular tests, extensive molecular epidemiological studies represent an important first step in the elucidation of their biological heterogeneity and varying virulence properties, as well as antiretroviral drug resistance. Currently, it remains unclear whether differences in disease progression exist between HIV-2 subtypes or CRFs. 

HIV-2 is comprised of eight different groups (A-H). Two major HIV-2 groups, groups A and B, were generated by two independent transmission events involving fuliginous mangabeys infected with the Taï Forest in Côte d’Ivoire, which appear to be linked with the vast majority of cases identified so far. Seven other HIV-2 groups have been described but each has only been identified in just one patient. In addition, these two groups have a different pattern of circulation in West Africa: Group A is widely present in all areas, while Group B is mainly located in Côte d’Ivoire, Ghana, Burkina Faso, and Mali [14]. The other groups were reported only in a few individuals. Groups C, D, E, and F were located in Sierra Leone and Liberia, and Group G in Côte d’Ivoire [35,36,37]. No subtypes have been formally described but some preliminary data suggest that HIV-2 Group A may be divided into two distinct subtypes with distinct geographical origins. Unlike HIV-1, only two recombinant forms have been described, labelled as CRF01_AB and the unique recombinant form, obtained by sequencing the entire genome of the virus obtained from an infected Japanese individual and two Nigerian patients most likely infected in their country of origin [38,39,40,41,42,43,44,45,46]. 

This low number of reported recombination events can be explained by two factors: (i) the low prevalence and lower transmissibility of the virus, resulting in a low number of patients infected with distinct strains of HIV-2, and (ii) the absence of the described HIV-2 subtypes and the high genetic distance between HIV groups that make such recombination events less frequent. In fact, the two formally described recombinant forms of HIV-2 correspond to intergroup recombination events. Three intergroup recombinants for HIV-2 have also been identified and one of them has recently been described as transmissible; however none of them have reached CRF status at this time [47]. The first recombinant form of intergroup HIV-2 was identified in 1994 by a patient sampled in 1990 in Abidjan, in the Ivory Coast [37]. This strain, called isolating 7312 A, has been classified as Group B for gag and pol regions, and as Group A for env. This strain remained the only unique recombinant form (URF) ever described for HIV-2 until 2008 when a second URF was identified by a patient sampled in 2003 in Douala, Cameroon [38]. In 2010, complete genome sequencing was obtained in three of the five new HIV-2 infection patients identified in Japan, including a Japanese patient infected in Japan and two Nigerian patients most likely infected in their home country, and revealed that these viral strains have the same recombination pattern as the 7312A. Therefore, this recombinant form has been confirmed as the first circulating recombinant form (CRF) for HIV-2, known as CRF01_AB, from the Los Alamos national laboratory [38].

**Figure 1:**
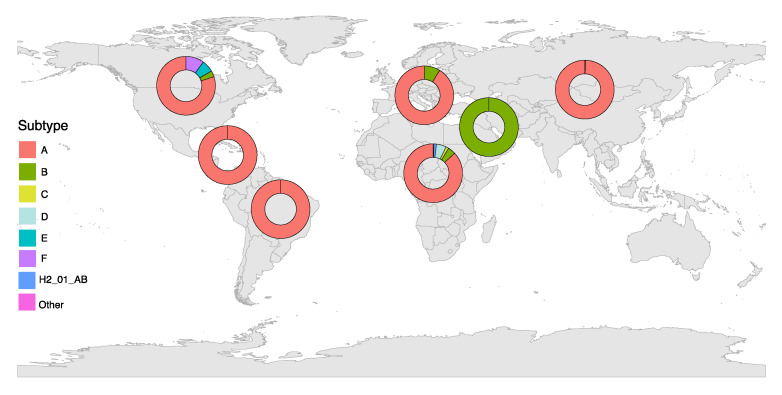
HIV-2 subtype distributions worldwide based on the frequency in the HIV Los Alamos Database (available from https://www.hiv.lanl.gov/content/sequence/HIV/mainpage.html, last accessed on 12 September 2021).

HIV-2-subtype A accounts for the majority of HIV-2 infections and is the predominant genotype in Guinea-Bissau and Europe (Figure 1) [44,47,48,49].

Group A and B show two distinct distributions throughout West Africa, as Group A is widely present throughout the area, but Group B is mainly located in Côte d’Ivoire, Ghana, Burkina Fasso, and Mali (Figure 1). 

Some survey studies have reported a decrease in HIV-2 prevalence in Senegal, Gambia, and Guinea-Bissau [2], showing that HIV-2 prevalence almost halved between 1987 and 2007 in Guinea-Bissau. On the basis of these results, Fryer et al. used a mathematical model to predict the dynamics of HIV-2 infection in a rural region of Guinea-Bissau, specifically Caió. This model predicted a rapid decline in HIV-2 prevalence, with an expected incidence of less than 0.1% of new infections by 2050, an end to new infections around 2043, and a complete extinction around 2068 [22]. As shown in several studies, competition from HIV-1 seems to contribute only slightly to the decline of HIV-2 [47]. In conclusion, the data that allow for the prediction of the extinction of an HIV-2 epidemic are mainly based on Guinea-Bissau, although some data from France and Portugal may also suggest a global decrease. Available data on the epidemiology in Africa is often based on less specific tests that can misclassify certain HIV infections and obfuscate HIV-2 prevalence data [47]. In this sense, new studies on HIV-2 prevalence in recent decades in other West African countries appear to be crucial in order to understand and predict the burden of the disease.

## 4. Natural History Clinical Manifestations

Although the modes of transmission for HIV-2 are the same as those for HIV-1, such as sexual contact, blood exposure (blood transfusion and shared needles), and perinatal transmission, HIV-2 has a lower infectivity than HIV-1 both through sexual and mother-to-child routes, probably due to its lower RNA viral load in infected individuals that could, in part, explain the difference in the pathogenicity between the two viruses [2,50]. In fact, sexual transmission is reduced by 5–9 times and vertical transmission by 10–20 times, compared to HIV-1 [51]. 

Compared to HIV-1, HIV-2 infection may have a longer asymptomatic latency period, low levels of plasmaviremia, higher CD4 T cell counts, slower CD4+ T cell exhaustion, and slower progression to AIDS [50,52,53,54,55,56,57,58,59]. Several studies indicate that only 15–25% of HIV-2 infections will progress to AIDS if following a natural course of disease, with an estimated average of 15–20 years in the absence of antiretroviral therapy [50] (Table 1).

The identification of unique immune signatures associated with HIV-2 pathogenesis can therefore provide therapeutically useful information regarding the management of HIV infection. Individuals with HIV-2 show clinical signs, symptoms, and opportunistic infections (OI) similar to those observed with HIV-1 (wasting syndrome, toxoplasmosis, cytomegalovirus disease, cryptosporidiosis, esophageal candidiasis, cerebral Mycobacterium avium diffuse intracellular, cryptococcal, tuberculosis, bacterial pneumonia, Kaposi sarcoma, and AIDS dementia complex) [59,60,61,62]. However, most people with HIV-2 infection never progress towards AIDS-free survival [63]. 

The rate of progression to AIDS in HIV-2 patients is variable, as some subjects develop advanced immunodeficiency and AIDS-related complications in a similar way to HIV-1 infection, while others have normal survival or progress very slowly [64,65]. These latest observations led some authors to speculate that HIV-2 could prevent subsequent HIV-1 infection. HIV-1 and HIV-2 co-infection may occur, which is believed to delay the progression of HIV-1 disease; however, a previous HIV-2 infection does not appear to protect against HIV-1 infection [65]. 

Differences in the natural history of HIV-1 and HIV-2 infection may result from host or viral factors, or, more likely, from a combination of the two. Although there are clear immune correlates of HIV-2 non-progression, such as the gag-specific CD8+ T cell response [52], other studies suggest that specific viral factors could play an important role [52]. HIV-2 causes a powerful, largely neutralizing antibody response in most infected people, leading to the hypothesis that the HIV-2 envelope may be one of the key factors explaining the reduced pathogenicity of HIV-2. However, the structure of HIV-2 gp120 has recently been determined and has shown notable similarities to HIV-1 gp120 [52]. This observation induced researchers to evaluate other viral factors involved in the origin of the clinical differences between HIV-1 and HIV-2. The Nef accessory protein is one of the potential contributing factors, as it is relevant in the replication and pathogenesis of HIV and SIV, and functional differences between the HIV-1 and HIV-2 Nef protein have been reported [52]. Recently researchers, in fact, suggest that low viral load and delayed disease progression are significantly linked to Nef deletions in both humans and primates [55]. 

## 5. Testing

Testing for HIV-2 infection is recommended by the CDC for individuals who are at risk based on exposure history or for those with an illness that suggests HIV infection but whose HIV-1 shows an unusual indeterminate result. Correct diagnosis of HIV-2 infections is clinically important given the varying clinical courses of these infections and because some antiretroviral agents effective against HIV-1 are not effective against HIV-2. In the USA, a testing algorithm is used to distinguish HIV-1 and HIV-2 [65,66], in which diagnostic tests, to detect HIV-2 infection, use fourth-generation immunoassays that incorporate specific antigens to detect antibodies directed against both HIV-1 and HIV-2. When HIV-1/HIV-2 immunoassays are repeatedly reactive and positive for both the HIV-1 and HIV-2 antibody (so-called HIV untypable or undifferentiated), the WHO and CDC also recommend the use of specific testing for HIV-2 for specimens with negative or indeterminate HIV-1 western blot results, such as nucleic acid-based differential testing. However, due to the absence of the US Food and Drug Administration (FDA)-approved nucleic acid test (NAT) confirming HIV-2 infection, samples are not clearly addressed in the algorithm [66].

Very recently, a novel qualitative amplification NAT for plasma, serum, and dried blood spot specimens has been developed. The automated cobas HIV-1/2 Qual test combines nucleic acid purification with real-time PCR, discriminating between HIV-1 and HIV-2, thus resulting in suitable confirmation of HIV-2 infection in the HIV testing algorithm [67].

In Japan, the guidelines recommend that HIV-2 infection can be confirmed using WB after a positive screening antibody/antigen test result. However, WB might show false-negative results and cross-reactions between HIV-1 and HIV-2, similar to NAT, although less than WB, thus numerous HIV-2-specific in-house NAT have been proposed and compared [68].

An in-house nested HIV-2 PCR assay targeting the 5′-long terminal repeats (5′-LTR) region has been also used as a confirmatory test and ensured HIV-1/2 differentiation in a South African public laboratory [69]. Moreover, a quantitative assay of HIV-2 RNA (HIV-2 Real Time RT-PCR Kit, Lfe River™, Shanghai ZJ Bio-tech Co., Ltd., Shanghai, China) has been validated for in vitro diagnostics [43].

## 6. Treatment and Resistance

At present, the scientific knowledge indispensable for achieving the targets identified by the WHO for its ambitious 90-90-90 project is still lacking for HIV-2 [3,24]. Even the evidence available in the field of diagnostics and therapy is currently inadequate to define with certainty the most effective management and treatment pathways, and is partly due to evidence gathered in the HIV-1 context. The establishment of evidence-based recommendations will only be possible on the basis of an accurate and systematic collection of information related to the management of HIV-2, which is currently deficient. In particular, updated clinical trials are needed that define the clinical criteria for the initiation of antiretroviral therapy and that evaluate the most appropriate first and second-line treatment regimens [24,70]. The study of the problems inherent in treatment failure in patients with HIV-2 infection requires in-depth studies that include data on the adequate management of resistances and the drugs available to combat them. Finally, it is essential to implement diagnostic resources for monitoring patients during treatment [3,24].

Currently, treatment for HIV-2-infected patients is more limited than for HIV-1, for which most antiretroviral compounds have been designed [10,24,70]. Some antiretrovirals are not active against HIV-2, including non-nucleoside reverse transcriptase inhibitors (NNRTI) such as efavirenz and nevirapine, due to the large extent of polymorphisms. Among protease inhibitors (PIs), only darunavir, lopinavir, and saquinavir are fully active. Atazanavir, fosamprenavir, indinavir, nelfinavir, and tipranavir have reduced antiretroviral activity against HIV-2 [24,70,71,72,73,74,75,76,77,78,79]. Effective drugs against HIV-2 are reported in Table 2. In particular, recent evidence has confirmed the potency of integration inhibitors on wild HIV-2 clinical isolates, suggesting that integrase strand transfer inhibitor (INSTI)-based combinations are effective and safe treatment options for these individuals [24,70,80,81].

On the basis of this evidence, current recommendations for the treatment of HIV-2 in well-resourced settings suggest to treat patients diagnosed with HIV-2 with a single-tablet regimen (STR), including an INSTI plus two nucleoside reverse transcriptase inhibitors (NRTIs) as a starting treatment (i.e., abacavir/lamivudine/dolutegravir or tenofovir alafenamide/emtricitabine/bictegravir) in naive non-pregnant adults.

Alternative suggested STR regimens include tenofovir alafenamide/emtricitabine/cobicistat/darunavir and tenofovir alafenamide/emtricitabine/cobicistat/elvitegravir.

Available once-daily multi-tablet regimens include abacavir/lamivudine plus raltegravir, tenofovir alafenamide/emtricitabine plus darunavir and ritonavir, tenofovir alafenamide/emtricitabine plus darunavir/cobicistat, tenofovir alafenamide/emtricitabine plus dolutegravir, or tenofovir alafenamide/emtricitabine plus raltegravir [10,24,70]. A single-tablet regimen containing elvitegravir, cobicistat, emtricitabine, and tenofovir disoproxil fumarate was also proposed [82].

In the case of pregnant women affected by HIV-2, the initial regimen should consider only safe drugs for mothers and children: the suggested regimens are built with a backbone of abacavir/lamivudine (if HBsAg and HLA-B*5701 tested negative) or tenofovir disoproxil fumarate/emtricitabine plus a raltegravir or ritonavir-boosted darunavir [10,24,70]. Bictegravir is not evaluated in women of child-bearing potential and pregnant women. Elvitegravir/c is not recommended for use during pregnancy and, regarding dolutegravir, prescribers should consider the child-bearing potential in women and be mindful of the recommendations of medical agencies for drug safety [24].

In patients with previous HIV-2 treatment failures and in experienced patients, antiretroviral drugs should be considered if their potency has not been impaired by the emergence of drug resistance. In cases of virological failure, however, drug resistance assays for HIV-2 are not commercially available in many countries, making it difficult to select an alternative treatment regimen [10,24,70].

The post-exposure prophylaxis (PEP) offered after HIV-2 exposure is based on the prescription of tenofovir disoproxil fumarate/emtricitabine plus raltegravir [10,24,70].

In poor-resource settings, not all active therapeutic options are available and the risk of suboptimal treatments is frequent, as evidenced by the data obtained from some real-life studies: for example, in a recent prospective, longitudinal observational cohort study performed by the Senegalese national AIDS program, only 88.1% of patients enrolled had a HIV-2-RNA suppressed and 56% showed a multiclass drug resistance profile, while 15% experienced virologic failure with no known pattern of resistance mutations [83].

Therapeutic management based on viral load monitoring and drug resistance tests is of great importance to avoid the emergence of complex resistance models. Resistance-associated mutations against antiretrovirals may be selected in HIV-2-infected patients while on therapy but data on HIV-2 resistance are scarce. A European consortium has compiled a list of drug resistance mutations, which appears to be regularly updated (HIV-2EU HIV-GRADE internet tool) and several algorithms are freely available online to interpret genotypic resistance to HIV-2 drugs, such as the Stanford HIV drug resistance database (http://www.hivdb.stanford.edu (accessed on 1 September 2021)) and German HIV-2 drug resistance analysis (http://www.hiv-grade.de (accessed on 1 September 2021)) [84,85]. Three key mutations of resistance, namely K65R, Q151M, and M184V, that occur in HIV-1, were frequently described in patients infected with HIV-2A [85,86,87,88,89,90,91]. Moreover, K65R and Q151M generate a high level of resistance for most NRTI drugs, leading to a high level of cross-resistance [89]. Regarding PI drugs, V47A is very common in the case of treatment failure with lopinavir, while V47A, I82F, and I54M have been described to cause a high level of phenotypic resistance to lopinavir and I54M for darunavir [75,76].

Despite the fact that INSTI-based regimens are an effective therapeutic resource, it is important to underline that mutations in INSTI resistance have led to being frequently selected in failing patients [80,81].

Finally, it is important to underline that, to date, only three trials on the HIV-2 topic are in progress, currently carrying out a search on the clinicaltrial.gov registery. In particular, the topics currently covered by these active recruiting trials are regarding (I) the clinical and immuno-virological response to antiretroviral treatment (NCT04658329); (II) the mother-to-child transmission of HIV 1/2 and its prevention (NCT03235310); and (III) prospective non-interventional observations of adult chronic HIV-1/2-infected patients who initiate antiretroviral therapy in routine care (NCT04888754) [92,93,94]. Therefore, in light of these evidences, it is confirmed that research on the topic of HIV-2 continues to be poor in contributions, despite the need to acquire new data from large clinical trials.

## 7. Conclusions

HIV-2 currently remains a neglected disease, apparently limited to specific geographic areas but which nevertheless involve significant percentages of the resident population. Scientific knowledge on the subject is progressing much slower than that on HIV-1 and the resources available for management at the moment are extremely limited. A goal for 2030 advocated by the scientific community is to move this disease out of oblivion and make it a key topic of scientific research. In particular, the main objective is to reach the same goals already indicated for HIV-1 but rather for HIV-2 within the great WHO project of 90-90-90 [3].

HIV-2 infection alone or as a co-infection with HIV-1 should be excluded at least once in all HIV-related people. This should be emphasized in the face of atypical HIV serological profiles, immunovirological disconnection (loss of CD4 T cell count and undetectable HIV-1 viremia), and/or high epidemiological risks. Superinfection with any variant of HIV can occur in people infected with the other variant since there is no cross-protection.

A surveillance system based on the molecular epidemiology of HIV-2 is becoming critical due to the increased migration from endemic areas, such as African countries, to Europe and especially to Italy, which is the first major landing spot for immigrants. Strengthening the genomic monitoring at a large scale appears to be challenging and crucial in order to determine the pattern of the spread of HIV-2 from endemic African countries to Europe, South America, North America, and Asia. In order to plan the best therapeutic treatment, a correct diagnosis of HIV-2 infection is essential. Therefore, it is important to use valid diagnostic tools, such as real-time quantitative PCR tests, to determine the viral load of HIV-2; to conduct a phylogenetic analysis to identify the type of HIV-2 in infected patients; and to monitor the origin of the infection and the spread of this neglected virus worldwide.

## Figures and Tables

**Table 1:** Comparison between HIV-1 and HIV-2 [4,13,19,20,21,22,23].

	HIV-1	HIV-2
Origin	Chimpanzee	S. Mangabey
Genetic diversity	High	Low
**Viral Characteristics**		
Infectivity	High	Low
Virulence	High	Low
Pathogenicity	High	Low
**Clinical characteristics**		
Illness	Majority develop AIDS	Majority LTNPs ≅25% develop AIDS
Time to develop AIDS (without treatment)	<10 years	>20 years
Opportunistic infections	Associated with similar opportunistic infections	
**Epidemiology**		
Routes of transmission	Transmitted through the same routes	
Vertical transmission	≅40%	≅4%
Geographical spread of the disease	Global	Endemic in West Africa Sporadic in other countries

**Table 2:** Overview of principal anti-retroviral compounds not-active/active against HIV-2 [10,24,70].

Class	Antiretroviral Drugs: HIV-2 Activity	
	Effective	Ineffective
***NUCLEOS(T)IDE ANALOGS***	*tenofovir, lamivudine, emtricitabine, abacavir, islatravir*	
***NON-NUCLEOSIDE ANALOGS***		*nevirapine, efavirenz, rilpivirine, etravirine, doravirine*
***PROTEASE INHIBITORS***	*lopinavir, darunavir, saquinavir*	
***INTEGRASE INHIBITORS***	*raltegravir, elvitegravir, dolutegravir, bictegravir, cabotegravir*	
***ENTRY INHIBITORS***	*maraviroc (CCR5 antagonist - in case of CCR5 tropism of the virus), ibalizumab*	*enfuvirtide (fusion inhibitor), fostemsavir*
***MATURATION INHIBITORS***		*bevirimat*
